# Acute Respiratory Failure Due to Mucous Plug in a Patient With Tropical Spastic Paraparesis

**DOI:** 10.7759/cureus.72812

**Published:** 2024-11-01

**Authors:** Hamza Hamza, Noah Silverstein, Samy I McFarlane, Hatim Idris, Daniel J Ouyang

**Affiliations:** 1 Internal Medicine, State University of New York Downstate Health Sciences University, Brooklyn, USA; 2 Cardiology, North Shore University Hospital, Long Island Jewish Medical Center, Long Island, USA; 3 Internal Medicine, Downstate-Health Science University, Brooklyn, USA; 4 General Medicine, University of Medical Sciences and Technology, Khartoum, SDN

**Keywords:** human t-cell lymphotropic virus type 1, mucous plug, respiratory failure, sepsis, tropical spastic paraparesis

## Abstract

Tropical spastic paraparesis (TSP) or human T-lymphotropic virus type 1 (HTLV-1) myelopathy is a chronic inflammatory disease of the spinal cord caused by HTLV-1. It usually presents as a disease of the lower extremities, characterized by paralysis and sensory loss. HTLV-1 is common. However, only a small fraction of those infected actually experience clinical symptoms. Respiratory complications are rare; this case discusses an uncommon presentation of acute respiratory failure in a patient with TSP. A bedridden 73-year-old female with HTLV-1-associated TSP, presenting with progressive deterioration of motor and sensory functions in the lower limbs for 10 years, was admitted with sepsis due to recurrent urinary tract infection. On admission, she had hypotension with leukocytosis. She had initial stabilization but showed hypoxia and hypotension on day 5 with a chest X-ray showing total left lung collapse due to an obstructive mucous plug. She has been given hypertonic saline nebulizers and chest physiotherapy, following which there was a partial recovery; however, she then suffered a cardiac arrest that was followed by a tracheostomy for airway protection. In this case, the potential respiratory risk factors associated with TSP, such as failure to clear secretions properly, are underlined. Further research is needed with respect to respiratory muscle involvement in TSP patients and proactive airway management strategies. The tracheostomy intervention performed here only proves the need for addressing respiratory complications in this vulnerable population.

## Introduction

Tropical spastic paraparesis (TSP) is a chronic progressive inflammatory disease of the spinal cord. It causes spastic paralysis that results in loss of mobility in the lower extremities, loss of sensation, and urinary incontinence [1]. TSP is caused by human T-lymphotropic virus type 1 (HTLV-1). Although HTLV-1 affects 10-20 million people worldwide, clinical manifestation is rare [2]. In this report, we present a unique case of TSP leading to recurrent acute respiratory failure.

## Case presentation

**Figure 1:**
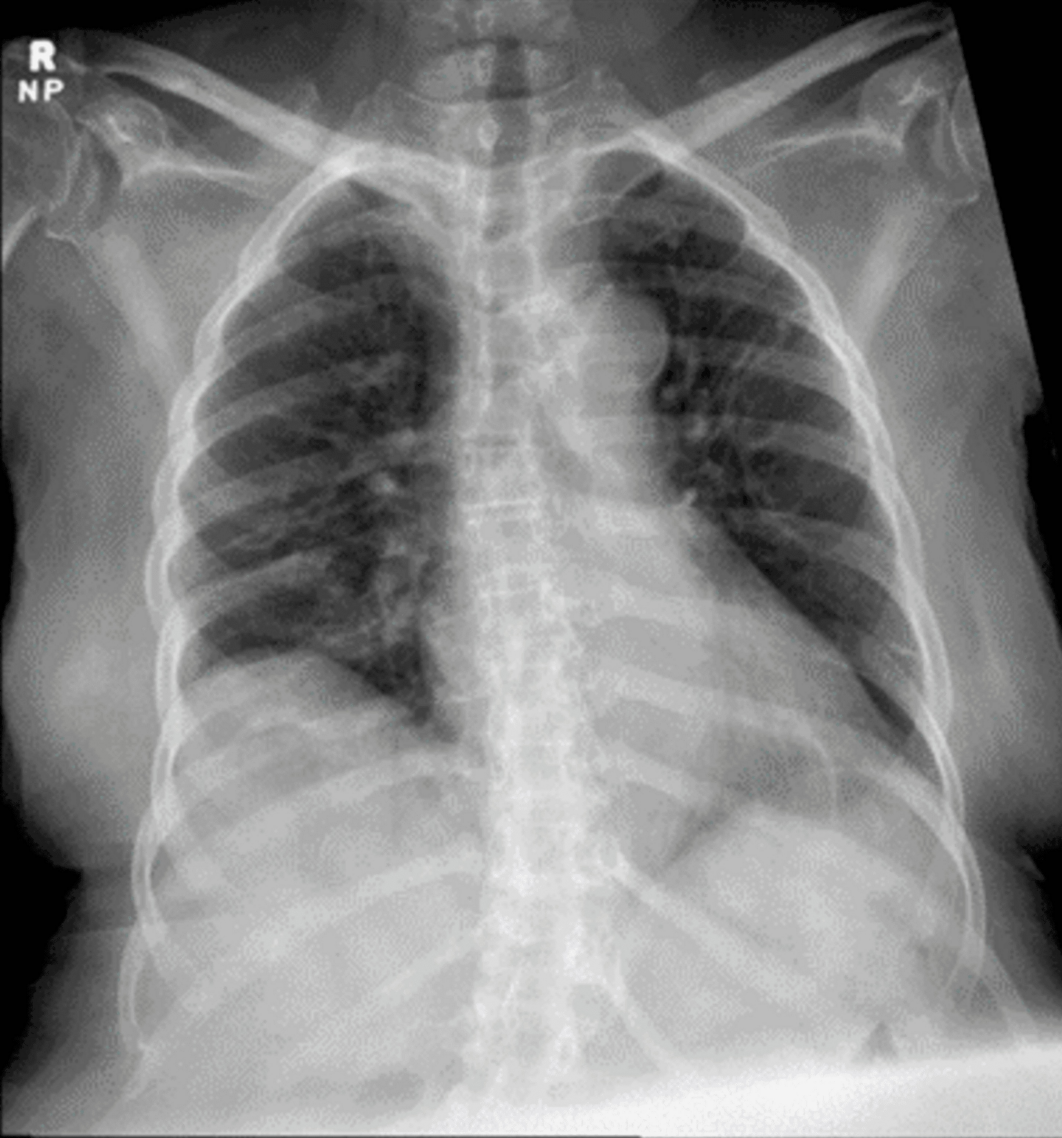
Anteroposterior CXR on the day of admission. CXR, chest x-ray

**Figure 2:**
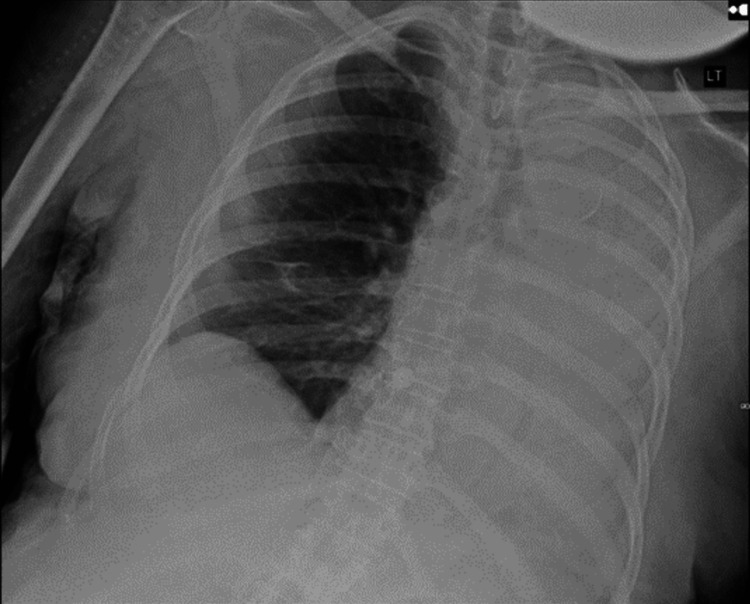
Anteroposterior chest x-ray five days post-admission. Complete opacification of the left lung field. CXR, chest x-ray

A 73-year-old bedbound woman with HTLV-1-associated TSP, presenting with progressive deterioration of motor and sensory functions in the lower limbs over the past 10 years, was admitted with sepsis secondary to a recurrent urinary tract infection. Her disease course was notable for the gradual loss of all motor function and sensation of lower extremities over a 10-year period. In the emergency department, the patient was found to be afebrile and hypotensive (blood pressure 87/59 mmHg), with a heart rate of 94 beats/minute and SpO_2_ of 98% on room air. Laboratory results were significant for a leukocytosis of 25 x 10^9^/mL with a neutrophilic predominance, lactate level was 3.0 mmol/dL, and creatinine of 1.68 mmol/dL. A urinalysis was remarkable for leukocytes and nitrites. A chest x-ray (CXR) (Figure 1) was unremarkable. She was resuscitated with intravenous (IV) fluids and antibiotics on admission. On hospital day 5, the patient developed a sudden hypoxic episode with 71% SpO_2_, associated with hypotension of 89/54 mmHg, requiring escalation of oxygen therapy. A CXR (Figure 2) was obtained, which showed complete opacification of the left hemithorax compatible with total left lung collapse, consistent with clinical and radiological diagnosis of obstructive mucous plug in the left mainstem bronchus. She was placed in a right lateral decubitus position and treated with interval hypertonic saline nebulizers, albuterol nebulizers, and chest physiotherapy. Subsequent daily CXRs showed improvement with decreased opacification of the left lung. On day 8, the patient became hypoxic and suffered a cardiac arrest. During intubation, copious secretions were suctioned. The patient was transferred to the Medical Intensive Care Unit (MICU) and ultimately underwent a tracheostomy due to the inability to protect the airway.

## Discussion

This case documents a unique presentation of acute respiratory failure due to a mucous plug, without apparent causes other than the TSP, due to the patient's inability to cough and clear her upper airway. While most TSP patients present only with lower extremity motor and sensory dysfunction, we are not aware of any documented cases demonstrating the involvement of the diaphragm or respiratory musculature. Although most patients with TSP usually experience motor and sensory dysfunction of the lower extremities, this case points out the important recognition of respiratory complications, including involvement of the diaphragm and musculature of respiration that can occur. Other conditions with neuromuscular impairment, like amyotrophic lateral sclerosis (ALS) and spinal cord injuries, have also reported similar cases. For example, studies undertaken by Neidermeyer et al. (2019) and Mayaux et al. (2019) reported that patients with severe ALS are often subject to respiratory failure precipitated by the retention of secretions, culminating in atelectasis and pneumonia [3,4]. Various studies discussed respiratory complications in patients with various forms of paralysis and indicated that an impaired cough reflex with poor airway clearance may lead to significant morbidity, including recurrent infections and respiratory failure [5-7]. These findings run parallel with our case and underline the importance of vigilance in monitoring respiratory function in TSP patients.

In the absence of any documented respiratory failure that could directly be attributed to TSP in the current literature, other complications arising from HTLV-1 infection, which include ALS-like disorder and different types of myopathy, have shown possibilities of presentation of respiratory failure [8,9]. This lack, therefore, signifies a dire need for more investigation of potential respiratory complications in TSP. The mechanisms leading to these complications should be understood. For instance, either the degeneration of the motor neurons or the muscle fibers could be a causative factor in reducing respiratory muscle strength and endurance, thus causing respiratory insufficiency.

These relationships need further investigation regarding the risk of respiratory complications in TSP patients, drawing from established knowledge about respiratory complications associated with other diseases caused by HTLV-1. The better one understands these mechanisms, the greater the diagnostic precision and monitoring of patients will be, leading to improved management strategies and outcomes for people affected by TSP and its complications.

The above insights emphasize the importance of incorporating respiratory management into the care plan of patients with TSP. Proactive measures would include the regular assessment of pulmonary function, early mobilization, and interventions such as chest physiotherapy, which may reduce the risk of respiratory failure. This will require additional research to understand the spectrum of respiratory dysfunction occurring in TSP and to devise specific strategies that may help improve outcomes and quality of life in patients.

## Conclusions

The potential development of respiratory muscle failure warrants further research in patients with TP in order to improve the care of this vulnerable population and prevent potentially life-threatening respiratory arrest as occurred in our patient twice. Our report also highlights the successful intervention in this case with a tracheostomy that bypasses the upper airway and provides an opportunity for better management and suction of airway secretion, thus preventing aspiration and upper airway obstruction.
